# Molecular Identification of Necrophagous Muscidae and Sarcophagidae Fly Species Collected in Korea by Mitochondrial Cytochrome c Oxidase Subunit I Nucleotide Sequences

**DOI:** 10.1155/2014/275085

**Published:** 2014-05-28

**Authors:** Yu-Hoon Kim, Sang Eon Shin, Chan Seon Ham, Seong Yoon Kim, Kwang Soo Ko, Tae-Ho Jo, Gi Hoon Son, Seong Hwan Park, Juck-Joon Hwang

**Affiliations:** ^1^Department of Legal Medicine, Korea University College of Medicine, Inchonro 73, Seongbukgu, Seoul 136-705, Republic of Korea; ^2^DaejeonDaejeon Institute of National Forensic Service, 1524 Yuseongdae-ro, Yuseong-gu, Daejeon 305-348, Republic of Korea; ^3^Department of Science Education, Chinju National University of Education, 3 Jinyangho-ro 369 gil, Jinju 660-756, Republic of Korea

## Abstract

Identification of insect species is an important task in forensic entomology. For more convenient species identification, the nucleotide sequences of cytochrome c oxidase subunit I (COI) gene have been widely utilized. We analyzed full-length COI nucleotide sequences of 10 Muscidae and 6 Sarcophagidae fly species collected in Korea. After DNA extraction from collected flies, PCR amplification and automatic sequencing of the whole COI sequence were performed. Obtained sequences were analyzed for a phylogenetic tree and a distance matrix. Our data showed very low intraspecific sequence distances and species-level monophylies. However, sequence comparison with previously reported sequences revealed a few inconsistencies or paraphylies requiring further investigation. To the best of our knowledge, this study is the first report of *COI* nucleotide sequences from *Hydrotaea occulta, Muscina angustifrons, Muscina pascuorum, Ophyra leucostoma, Sarcophaga haemorrhoidalis, Sarcophaga harpax*, and *Phaonia aureola*.

## 1. Introduction

The postmortem interval (PMI) is a key piece of information that needs to be determined in the investigation of a death. In fresh bodies, early postmortem changes such as body cooling, rigidity, and lividity are used for the estimation of PMI [1]. In putrefied bodies, however, these early changes cannot be used for PMI estimation, and it is not possible to estimate PMI from the degree of putrefaction [1]. As a result, PMI estimation in putrefied bodies is one of the most difficult tasks for forensic scientists and pathologists.

Many kinds of arthropods, especially insects belonging to the orders Diptera (flies) and Coleoptera (beetles), are attracted to the bodies of dead animals. Flies, particularly blow flies (Family Calliphoridae), are typically the first to arrive and oviposit into animal carcasses [2]. In addition to blow flies, 2 other families, Muscidae (house flies and allies) and Sarcophagidae (flesh flies), are important in forensic entomology. Although house flies are not commonly attracted to putrefied meat as blow flies and flesh flies are, they are often important indicators of PMI particularly in indoor deaths [2]. When larvae or pupae in various stages of development are collected from the site of investigation and the growth rates of samples are known, an approximate time of oviposition or larviposition can be estimated [3]. Species identification is essential for determining growth rates, as these rates are species-specific [2]. Therefore, species identification is a key step in estimating the PMI from entomological evidence. The traditional species identification method is dependent on the morphological features of insects and is not easily applicable to immature samples such as eggs, larvae, and pupae [4–9]. Moreover, only a few expert taxonomists specialize in forensically important insect species, not only in Korea but also worldwide. DNA-based approaches have been developed in an effort to improve accessibility to methods of species identification. Sperling et al. developed a method to identify 3 forensically important fly species by using the mitochondrial cytochrome c oxidase subunit I (*COI*) gene and its flanking loci [10]. Although mitochondrial* COI* nucleotide sequence analysis frequently yields species-level or even genus-level paraphylies in forensically important flies, this locus is still used as the standard method of identification [11, 12]. Two previously reported studies have used the full-length DNA of the* COI* gene for Calliphoridae species in Korea [13, 14]. However, there has been little effort to characterize the* COI* haplotypes of Korean Muscidae and Sarcophagidae fly species. This study examined the full-length nucleotide sequences of the* COI* gene of 10 Muscidae and 6 Sarcophagidae fly species collected in Korea.

## 2. Materials and Methods

### 2.1. Sample Collection and Preparation

Fly samples were collected between 2004 and 2008 in Seoul, Guri, Pyeongtaek, and Jeju Island regions of Korea by using insect nets or traps with pork liver bait. Because fly collection was performed in private lands except for in Jeju Island, no specific permission was required in Seoul (Korea University College of Medicine), Guri (JJH's private residence), and Pyeongtaek (PWG Genetics Company). The GPS information for the collection sites in Seoul, Guri, and Pyeongtaek is 37.59,127.03, 37.58,127.11, and 37.05,126.97, respectively. For Jeju Island, we acquired permission from the Ministry of Environment of Korean Government. Pork liver bait and our collection method did not involve endangered or protected species. Species identification was performed by an expert dipterological taxonomist (Jo, TH) by using a dissecting microscope [4, 15–17]. Taxonomic information and the sample sizes of the flies analyzed are listed in Table 1. Flies were first frozen in liquid nitrogen, and the whole bodies were ground using a SKMILL-200 (Tokken, Chiba, Japan). Genomic DNA was extracted from the ground samples by using a QIAamp DNA Mini Kit (Qiagen, Hilden, Germany) according to the manufacturer's instructions.

### 2.2. Polymerase Chain Reaction (PCR) and Automatic Sequencing

Universal primer sequences for the* COI* gene were taken from the literature (Table 2) [13, 14, 24–26], and PCRs were performed using a 2720 Thermal Cycler (Applied Biosystems, Foster City, CA, USA). The PCR reaction conditions consisted of an initial denaturation step at 95°C for 11 min, followed by 35 cycles at 95°C for 30 s, 50°C for 1 min, and 72°C for 1 min, and then a final elongation step at 72°C for 15 min. Each reaction mixture was prepared using 50 ng of template DNA, 2.5 *μ*L 10× Amplitaq Gold Buffer, 0.5 U AmpliTaq Gold DNA Polymerase (Applied Biosystems, Foster City, CA, USA), 10 pmol (each) upstream and downstream primers, 62.5 nmol MgCl_2_, 5 nmol (each) dNTPs, and sterile distilled water to a final volume of 25 *μ*L. After purification of the PCR products, cycle sequencing reactions were performed according to the manufacturer's instructions using a BigDye v3.1 Cycle Sequencing Kit (Applied Biosystems, Foster City, CA, USA). The sequencing products were analyzed using an ABI 3730xl Genetic Analyzer (Applied Biosystems, Foster City, CA, USA). Assembled sequences were deposited into the NCBI GenBank database (JX861406–JX861482).

### 2.3. Phylogenetic Analysis and Sequence Comparison

Phylogenetic trees were generated for 2 fly families by using the maximum likelihood method with 1,000 replicates of bootstrapping based on the Tamura-Nei model using MEGA6 software [27]. Initial trees for the heuristic search were obtained by applying the neighbor-joining method to a matrix of pairwise distances estimated using the maximum composite likelihood (MCL) approach. To make a root for each tree,* COI* sequences for* Lucilia sericata* (NCBI accession number EU880212),* Calliphora vicina* (EU880188), and* Drosophila melanogaster* (NC_001709) were introduced as outgroup taxa. Average intraspecific and interspecific sequence distances were calculated for sequence comparison. Sequences obtained in this study were also compared to previously announced sequence data (Table 3).

## 3. Results

### 3.1. Nucleotide Sequence Distances

A pairwise percentage distance matrix of 10 Muscidae fly species is shown in Table 4. Because only 1 individual* COI* sequence was obtained for* H. occulta*, intraspecific variation was not estimated for this species. Interspecific distance was the lowest between* O. chalcogaster* and* O. leucostoma* (6.3%) and the highest between* Musca domestica* and* Phaonia aureola* (15.3%). Intraspecific distances were 0.3% or less.

A pairwise percentage distance matrix for the 6 Sarcophagidae fly species is shown in Table 5. Interspecific distance was the lowest between* Sarcophaga similis* and* Sarcophaga peregrina* (6.4%), whereas it was the highest between* Sarcophaga haemorrhoidalis* and* S. peregrina* (8.9%). Intraspecific distances were 0.3% or less.

### 3.2. Phylogenetic Analysis

**Figure 1:**
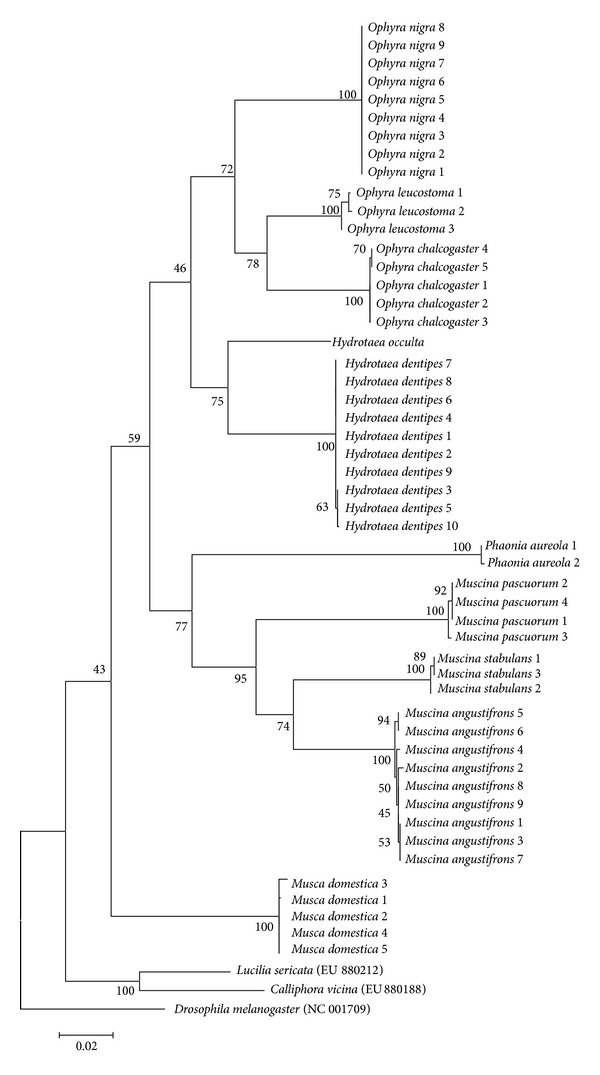
A phylogenetic tree was constructed for 10 Muscidae fly species by using the maximum likelihood method based on the Tamura-Nei model. The tree with the highest log likelihood (−8320.2383) is shown. The analysis involved 54 nucleotide sequences. All positions containing gaps and missing data were eliminated. There were a total of 1536 positions in the final dataset.* COI* nucleotide sequences of* Lucilia sericata* (EU880212),* Calliphora vicina* (EU880188), and* Drosophila melanogaster* (NC_001709) are included as outgroup taxa.

**Figure 2:**
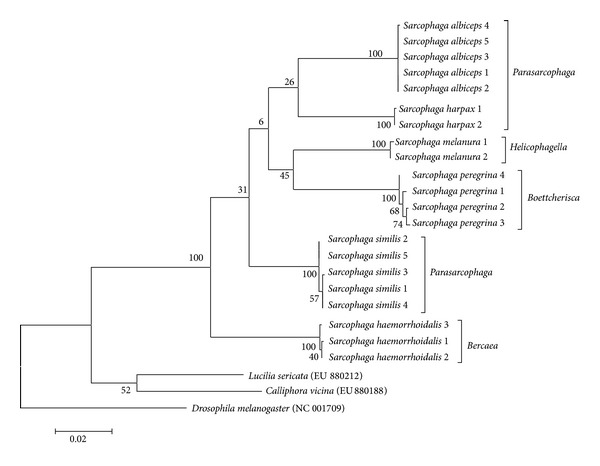
A phylogenetic tree was generated for 6 Sarcophagidae fly species by using the maximum likelihood method based on the Tamura-Nei model. The tree with the highest log likelihood (−5586.2586) is shown. The analysis involved 24 nucleotide sequences. All positions containing gaps and missing data were eliminated. There were a total of 1536 positions in the final dataset. A* COI* nucleotide sequence of* Lucilia sericata* (EU880212) is included as an outgroup.* COI* nucleotide sequences of* Lucilia sericata* (EU880212),* Calliphora vicina* (EU880188), and* Drosophila melanogaster* (NC_001709) are included as outgroup taxa. The taxa names in the italic grouping the external nodes mean the old genera of those species.

Maximum likelihood phylogenetic trees were generated from* COI* nucleotide sequences of 10 Muscidae and 6 Sarcophagidae fly species. All taxa were clustered according to species and genera, without any species- or genus-level paraphyly (Figures 1 and 2). Although a few internal nodes display low bootstrap values under 50%, every bootstrap value at the species level was 100%.

## 4. Discussion

As shown in Tables 4 and 5, Korean Muscidae and Sarcophagidae fly species showed average intraspecific sequence distances of 0.0–0.3%. The phylogenetic trees did not show any species-level paraphylies (Figures 1 and 2). Although our sampling was limited to a few areas of Korea in a relatively short period, these findings suggest that Korean Muscidae and Sarcophagidae fly species are identifiable using the* COI* nucleotide sequences.

In this study,* H. dentipes* showed intraspecific sequence distances of 0–0.1% (average 0.0%). The only previous* COI* sequence of* H. dentipes* (FJ025623) in the NCBI GenBank (Table 3) showed intraspecific distances of 3.5–3.6% from the conspecific sequences in this study [18]. According to Cognato, who reported intraspecific sequence distances of 0.04–3.5% in 8 fly species, this range of intraspecific distances (3.5–3.6%) may be valid and not a result of misidentification [28]. Further sampling from other geographic regions will be required, however, to confirm the variability of* COI* haplotypes of* H. dentipes*.

Because only 1* H. occulta COI* sequence was identified in this study, and there are currently no* COI* sequences from this species in the NCBI GenBank, it is impossible to determine the validity of this sequence. As expected, however,* H. occulta* formed a genus* Hydrotaea* clade with* H. dentipes* (Figure 1). Previously reported sequences from* H. cyrtoneurina*,* H. irritans*, and* H. dentipes* in the NCBI GenBank (Table 3) showed interspecific distances of at least 7.4% compared with the* H. occulta* sequence determined in this study [18].

*M. domestica*, the common house fly, exhibits a cosmopolitan distribution [6]. The* COI* gene has been widely studied in this species, and 28* COI* sequences of this species from the NCBI GenBank (Table 3) are highly homologous to conspecific sequences in this study (average distance = 0.2%) [19, 20].

As reported by Shinonaga, 5 species of the genus* Muscina* have been identified in Japan [6]. Three of these species were analyzed in this study. Of these,* M. stabulans* (stable fly) is the most forensically important species, and it is more often attracted to decaying animals than are other* Muscina* flies [6].

All 3* Muscina* flies showed very low intraspecific sequence distances (0.1–0.2%) and interspecific distances of at least 8.5%; hence, identification of Korean* Muscina* fly species was relatively straightforward. Compared to previously reported conspecific data, in this study,* M. stabulans* sequences were very similar to 2 previously reported conspecific sequences (EU627711 and AJ879595; sequence distance 0.1–0.3%) but very divergent from another reported sequence (EF531210; sequence distance 5.0–5.1%) [21]. Because only EF531210 is inconsistent with other conspecific sequences, the validity of this sequence should be reviewed by analysis of the voucher specimen and the morphological features used for identification. The* M. assimilis* sequence (EU627712) does not match any* Muscina* sequences reported in this study.

Three* Ophyra* species were analyzed in this study, each with low intraspecific distances and at least 6.3% interspecific distances. Therefore, identification of these 3 Korean* Ophyra* species is plausible. Compared to previously reported conspecific sequences, the* O. nigra* sequence obtained in this study was monomorphic with EU627714 (distance 0.3%), whereas* O. chalcogaster* showed distances of 1.2–1.3% from EU627715. Since the* O. leucostoma COI* gene has not previously been analyzed, conspecific comparison is not possible at this time. There are no nucleotide sequences in the NCBI GenBank database that match the* O. leucostoma* sequences reported in this study.

*S. haemorrhoidalis* showed a very low intraspecific average sequence distance (0.1%) and interspecific distances of at least 6.8% (Table 5). There are currently no other* COI* nucleotide sequences in the NCBI GenBank for this species name. However, a* COI* sequence of a synonymous species,* Sarcophaga africa* (GQ223343), is available [17]. Since the sequence distance between* S. haemorrhoidalis* and* S. africa* is only 0.8%, the DNA result also supports that they are conspecific.

**Figure 3:**
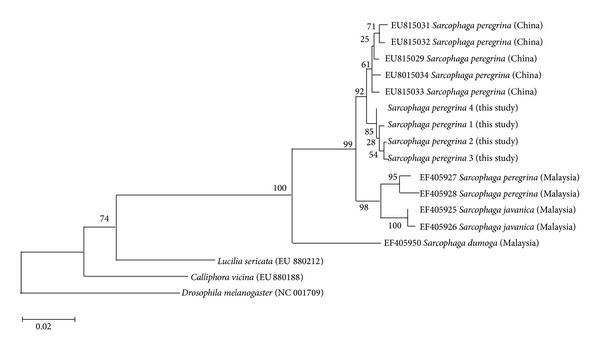
A maximum likelihood phylogenetic tree using data of the old genus* Boettcherisca* from this study (1–4) and the other authors' work based on the Tamura-Nei model. The tree with the highest log likelihood (−3420.3779) is shown. The analysis involved 17 nucleotide sequences. All positions containing gaps and missing data were eliminated. There were a total of 1076 positions in the final dataset.* COI* nucleotide sequences of* Lucilia sericata* (EU880212),* Calliphora vicina* (EU880188), and* Drosophila melanogaster* (NC_001709) are included as outgroup taxa.

*S. peregrina* sequences in this study showed a very low intraspecific average sequence distance (0.1%) and interspecific distances of at least 6.4% (Table 5). Because* S. peregrina* was once categorized in the old genus* Boettcherisca*, a phylogenetic tree was generated from* S. peregrina* sequences in this study and the* COI* sequences of old genus* Boettcherisca* submitted by other authors. The phylogenetic tree showed a species-level paraphyly of* S. peregrina*, with 2 Malaysian* S. peregrina* sequences, submitted by Tan et al., clustering with 2 Malaysian* S. javanica* sequences (Figure 3) [22]. Because these 2 Malaysian* S. peregrina* sequences are divergent from other conspecific sequences from Korea and China (sequence distance 2.4–3.0%), further consideration, such as a review of the voucher specimens, would be necessary.

*Sarcophaga melanura* showed a very low intraspecific average sequence distance (0.1%) and interspecific distances of at least 6.5% (Table 5). Compared with the 6 short* S. melanura COI* sequences shown in Table 3, the* S. melanura COI* sequences reported in this study showed intraspecific distances of only 0.0–0.7% [23].

Three species previously classified as the old genus* Parasarcophaga*, that is,* S. similis*,* Sarcophaga harpax*, and* Sarcophaga albiceps*, showed very low intraspecific average sequence distances (0.0–0.1%) and interspecific distances of at least 6.4% (Table 5). Compared with other conspecific species in the NCBI GenBank (Table 3),* S. albiceps* and* S. similis* showed intraspecific sequence distances of only 0.3–0.7% and 0.2–0.4%, respectively [22]. Additionally,* S. harpax* and its known sister species* S. dux* are closely related with sequence distances of 1.4–1.6% [22].

In conclusion, 10 Muscidae and 6 Sarcophagidae fly species collected in Korea were identifiable using* COI* sequence analysis. However, a few inconsistencies with previously reported sequences require further evaluation. To our knowledge, the present study provides the first report of the* COI* nucleotide sequences of* H. occulta*,* M. angustifrons*,* M. pascuorum*,* O. leucostoma*,* S. haemorrhoidalis*,* P. harpax*, and* P. aureola*.

## Figures and Tables

**Table 1:** Muscidae and Sarcophagidae fly species and sample sizes analyzed.

Family	Subfamily	Tribe	Genus	Species	Sample size
Muscidae	Muscinae	Reinwardtiini	*Muscina *	*angustifrons *	9
				*pascuorum *	4
				*stabulans *	3
		Azeliini	*Hydrotaea *	*armipes = occulta *	1
				*chalcogaster = Ophyra chalcogaster *	5
				*dentipes *	10
				*ignava = Ophyra leucostoma *	3
				*spinigera = Ophyra nigra *	9
		Muscini	*Musca *	*domestica *	5
	Phaoniinae	Phaoniini	*Phaonia *	*aureola *	2

Sarcophagidae	Sarcophaginae		*Sarcophaga *	*haemorrhoidalis = africa *	3
				*peregrina *	4
				*melanura *	2
				*albiceps *	5
				*harpax *	2
				*similis *	5

*This classification of Muscidae and Sarcophagidae is modeled on the basis of previous reports by Shinonaga  (2003) [15] and Kano et al.  (1967) [4] and by Pape (1996) [17], respectively.

**Table 2:** Universal primer sequences.

Name	Sequence	Binding site
F1	5′-CCTTTAGAATTGCAGTCTAATGTCA-3′	tRNA-cysteine
F2	5′-GGAGGATTTGGAAATTGATTAGTTCC-3′	220–245 on COI
F3	5′-CTGCTACTTTATGAGCTTTAGG-3′	1000–1022 on COI
R1	5′-CCTAAATTTGCTCATGTTGACA-3′	2–23 on COII
R2	5′-CAAGTTGTGTAAGCATC-3′	1327–1343 on COI
R3	5′-CCAAAGAATCAAAATAAATGTTG-3′	688–710 on COI

**Table 3:** Reference sequences from NCBI GenBank.

Family	Species name	NCBI accession number	Coverage on COI	Geographic region	Author	Reference
Muscidae	*Hydrotaea cyrtoneurina *	FJ025622	52–635 748–1454	Unknown	Kutty et al.	[18]
	*Hydrotaea dentipes *	FJ025623	48–635 748–1484	Unknown	Kutty et al.	[18]
	*Hydrotaea irritans *	FJ025624	2–635 748–1484	Unknown	Kutty et al.	[18]
	*Musca domestica *	EU814984–EU815009*	156–1268	Beijing, China	Chen, Q et al.	Unpublished
	*Musca domestica *	GQ465784	30–1524	Unknown	Wiegmann, BM	Unpublished
	*Musca domestica *	AY526196	1–1536	Brazil	de Oliveira et al.	[19]
	*Musca domestica *	FJ153278	1054–1539	Bangkok, Thailand	Preativatanyou et al.	[20]
	*Muscina assimilis *	EU627712	1–1536	Unknown	Meng, J et al.	Unpublished
	*Muscina stabulans *	EF531210	68–659 775–1446	Unknown	Petersen et al.	[21]
	*Muscina stabulans *	EU627711	1–1536	Unknown	Meng, J et al.	Unpublished
	*Muscina stabulans *	AJ879595	8–701	Parana, Curitiba, Brazil	Schuehli, GS et al.	Unpublished
	*Ophyra chalcogaster *	EU627715	1–1536	Unknown	Meng, J et al.	Unpublished
	*Ophyra spinigera = nigra *	EU627714	1–1536	Unknown	Meng, J et al.	Unpublished

Sarcophagidae	*Sarcophaga africa *	GQ223343	1–1539	Unknown	Stamper, T et al.	Unpublished
	*Sarcophaga dumoga *	EF405950	1–1534	Malaysia	Tan et al.	[22]
	*Sarcophaga javanica *	EF405925 EF405926	1–1534	Malaysia	Tan et al.	[22]
	*Sarcophaga peregrina *	EU815029–EU815034*	170–1277	Beijing, China	Chen, Q et al.	Unpublished
	*Sarcophaga peregrina *	EF405927 EF405928	1–1534	Malaysia	Tan et al.	[22]
	*Sarcophaga melanura *	AY315649	1027–1322	Unknown	Zehner et al.	[23]
	*Sarcophaga melanura *	HM037109	1049–1326	Xining, Qinghai, China	Cai, JF et al.	Unpublished
	*Sarcophaga melanura *	HM037110	1049–1326	Yinchuan, Ningxia, China	Cai, JF et al.	Unpublished
	*Sarcophaga melanura *	HM037111 HM037112	1049–1326	Shijiazhuang, Hebei, China	Cai, JF et al.	Unpublished
	*Sarcophaga melanura *	FJ746473	1047–1326	Lanzhou, Gansu, China	Cai, JF et al.	Unpublished
	*Sarcophaga albiceps *	EF405931 EF405932	1–1534	Malaysia	Tan et al.	[22]
	*Sarcophaga similis *	AY879256	304–855	Unknown	Song, Z et al.	Unpublished
	*Sarcophaga dux *	EF405937–EF405939	1–1534	Malaysia	Tan et al.	[22]

*EU815001 and EU815030 were excluded.

**Table 4:** Average pairwise percentage distances for 10 Muscidae fly species.

De	0.0									
Oc	7.2	N/A								
Do	11.5	11.4	0.2							
An	10.1	9.9	12.2	0.2						
Pa	12.1	11.8	14.3	10.2	0.1					
St	11.9	11.0	12.2	8.5	11.6	0.1				
Cg	9.1	8.7	10.9	11.7	12.8	12.7	0.0			
Le	7.7	8.2	10.6	10.4	11.8	12.0	6.3	0.3		
Ni	9.4	8.8	10.7	11.2	14.1	12.7	8.3	7.6	0.0	
Au	13.5	13.6	15.3	14.4	15.2	14.1	14.1	13.7	14.5	0.1

	De	Oc	Do	An	Pa	St	Cg	Le	Ni	Au

De = *H. dentipes*, Oc = *H. occulta*, Do = *M. domestica*, An = *M. angustifrons*, Pa = *M. pascuorum*, St = *M. stabulans*, Cg = *O. chalcogaster*, Le = *O. leucostoma*, Ni = *O. nigra*, Au = *P. aureola*, and N/A = not available.

**Table 5:** Average pairwise percentage distances for 6 Sarcophagidae fly species.

Hm	0.1					
Pg	8.9	0.3				
Me	7.6	6.8	0.1			
Al	8.2	7.3	6.8	0.0		
Ha	7.6	7.7	7.7	6.5	0.1	
Si	6.8	6.4	6.7	6.5	6.5	0.1

	Hm	Pg	Me	Al	Ha	Si

Hm = *S. haemorrhoidalis*, Pg = *S. peregrina*, Me = *S. melanura*, Al = *S. albiceps*, Ha = *S. harpax*, and Si = *S. similis*.
